# Relative Age Effect Analysis in the History of the Ballon d’Or (1956–2023)

**DOI:** 10.3390/sports12040115

**Published:** 2024-04-22

**Authors:** Miguel A. Saavedra-García, Miguel Santiago-Alonso, Helena Vila-Suárez, Antonio Montero-Seoane, Juan J. Fernández-Romero

**Affiliations:** 1 Grupo de Investigación en Ciencias del Deporte (INCIDE), Departamento de Educación Física y Deportiva, Universidade da Coruña, Oleiros, 15179 A Coruña, Spain; miguel.santiago@udc.es (M.S.-A.); evila@uvigo.es (H.V.-S.); antonio.montero.seoane@udc.es (A.M.-S.); juan.jose.fernandez@udc.es (J.J.F.-R.); 2 Departamento de Didácticas Especiales, Facultad de Educación y Ciencias del Deporte, Universidade de Vigo, 36005 Vigo, Spain

**Keywords:** football, performance analysis, talent development, Ballon d’Or, awards

## Abstract

Ballon d’Or is the most important individual award in football, and is a significant measure of excellence. From our knowledge, this is the first study that explored the relative age effect (RAE) throughout the history of the Ballon d’Or. A total of 1899 football players nominated for the award from the first edition in 1956 to the most recent edition (2023) were analyzed. To assess the RAE, the birthdate distributions were categorized into four trimesters. The comparison involved correcting for the uniform distribution using chi-square analysis, with Cramer’s V serving as a measure of effect size. Standardized residuals were computed to identify quarters that exhibited significant deviation from the expected values. Odds Ratio and 95% confidence intervals were used to identify discrepancies between trimesters. The results indicated a pronounced presence of an RAE at the global level. However, the longitudinal analysis revealed variations in the behavior of the RAE over time. In the initial decades, there is an overrepresentation of players born in the last months of the year. Subsequently, there is no discernible RAE. In the most recent decades, there has been a clear resurgence of RAE, with an overrepresentation of players born in the first quarters of the year.

## 1. Introduction

The relative age effect (RAE) refers to the phenomenon wherein individuals born closer to the cutoff date, for age-based selection in a competitive context, tend to have a significant advantage over their younger counterparts born away from the cutoff date [1,2]. In sports, the cutoff date is commonly set as the 1st of January in most competitions, giving players born in the first months of the year a sporting advantage (explained by the maturational profile associated with a late maturation of athletes born in the last months of the year) [3]. In most countries, the cutoff date for youth sports is the 1st of January [4], and in football, the Fédération Internationale de Football Association (FIFA) youth tournaments have also used this cutoff date to establish age groups since 1997 [5,6]. In professional football the cutoff date can vary between countries; for example, England uses August 1st [7]. Despite the literature acknowledging the differences between countries regarding their sport system and cutoff dates, FIFA’s categories represent a sound choice for football studies [8].

It is important to consider that the RAE is a statistical tendency and does not apply uniformly to every individual [9,10], while it can influence outcomes in terms of individual talents [11]. The relative age effect is particularly pronounced in activities like football, where age significantly influences eligibility and participation [12,13,14]. This effect is especially prominent among younger athletes [15,16] and extends to elite footballers. The primary explanation for RAE in football revolves around physical attributes, which hold greater significance than technical skills during early developmental stages. Moreover, the selection pressures faced by footballers at younger ages persist into senior elite levels [1,17,18].

While there is a considerable number of studies on RAE in football, ranging from older ones [19,20] to the most recent [8,21], it is infrequent to find publications that examine RAE in football over an extended period [7,17].

We identified longitudinal studies that include national team players [17] participating in FIFA tournaments, who face the extreme difficulty of selection in the current era due to the enormous competitive pressure that is increasing the RAE in football. However, we found no prior studies linking RAE to the nomination or awarding of individual prizes, such as the Ballon d’Or organized by France Football magazine. The Ballon d’Or is an annual football award, considered one of the most prestigious individual awards in this sport, recognizing the best male soccer player in world soccer since 1956 and the best female soccer player in world soccer since 2018. Introduced in 1956 by the chief editor of France Football, the Ballon d’Or was initially exclusive to European players playing in Europe. In 1995, a partnership between FIFA and France Football merged the FIFA World Player of the Year award with the Ballon d’Or. However, in 2016, France Football and FIFA separated their awards. FIFA introduced “The Best FIFA Men’s Player”, while France Football continued to present the Ballon d’Or. The Ballon d’Or is a significant measure of individual excellence in football. While there are very few studies about the Ballon d’Or, some characterize the nominated players [22], others examine the vote [23,24], or analyze the celebrities who have won it the most times [25]. However, from our knowledge, none of these studies examine the relative age effect.

This study aims to analyze the relative age effect in the history of the Ballon d’Or, examining its existence and evolution from the inaugural edition in 1956 to the present day (2023).

## 2. Materials and Methods

### 2.1. Data Collection and Participants

An empirical research design utilizing a descriptive observational strategy was employed [26]. The sample comprised all male footballers nominated for the Ballon d’Or from 1956 to 2023. Data were extracted from the official Ballon d’Or website (https://www.francefootball.fr/ballon-d-or/palmares/, accesed on 3 April 2022). The sample consisted of a total of 1899 athletes, with an average age of 27.09 ± 3.72 years.

### 2.2. Procedure and Data Analysis

We analyzed variables related to the year of the award, ranking, points (absolute and relative values), and date of birth. After acquiring the data, we derived the variable “quarter of birth” from the athletes’ date of birth. Similar to other authors [27,28,29], we considered the number of days in each quarter, providing a minor correction to the uniform probability distribution

$$X_{D}^{2}=\sum_{i=1}^{4}\frac{{\left(n_{i}-n.d_{i}\right)}^{2}}{n.d_{i}}$$

with $d_{1}=\frac{31+28.25+31}{365.25}$, $d_{2}=\frac{30+31+30}{365.25}$, $d_{3}=\frac{31+31+30}{365.25}$, and $d_{4}=\frac{31+30+31}{365.25}$ where $n_{i}$ is the observed frequency of births in trimester i. Thus, the values of $d_{1}$ to $d_{4}$ are 0.247; 0.249; 0.252; and 0.252, respectively. In this way, the selected players were categorized into birth quarters based on their month of birth (Q1: January, February, March; Q2: April, May, June; Q3: July, August, September; Q4: October, November, December). Additionally, the year was divided into semesters (S1: January to June, S2: July to December).

To investigate the presence of RAE among the best football players nominated for the Ballon d’Or from 1956 to 2022, chi-squared goodness-of-fit tests (χ^2^) were used to assess differences in observed and expected distributions across each birth trimester. Effect sizes for 3 degrees of freedom were calculated using Cramer’s V and were considered small for values between 0.06–0.17, medium for 0.18–0.29, and large for values higher than 0.29 [30]. Standardized residuals (post-hoc tests) were calculated to identify quarters significantly deviating from the expected values [31,32,33]. Values greater than 1.96 in absolute value are considered significant for the standardized residual. Positive values indicate an overrepresentation of births in a quarter of the year relative to the expected value, while negative values indicate an underrepresentation of births in a quarter of the year relative to the expected value. Finally, odds ratio (OR) and 95% confidence intervals were used to identify discrepancies between trimesters (comparing quartiles with each other, such as Q1 vs. Q2, Q1 vs. Q3, Q1 vs. Q4) and semesters (S1 vs. S2). The OR with thresholds 1.22, 1.86, and 3.00 served as benchmarks for small, medium, and large effect sizes, respectively [34]. All statistical analyses were performed using the SPSS 29 program, with the statistical significance level set at *p* < 0.05.

## 3. Results

RAE is significant in the history of the Ballon d’Or, as evident in Table 1. However, while it globally influences players who receive votes, it is not observed among players with the most votes. Despite a consistent bias with an overrepresentation of players born in the first months of the year, this bias is not observed among players who win or come close to winning the trophy. A small effect is observed in the overall sample, with a significant overrepresentation of athletes born in the first trimester and an underrepresentation of those born in the third trimester.

The evolution of RAE throughout the history of the Ballon d’Or indicates that in the first decades since its inaugural edition (the 1950s and 1960s), an inverse RAE could be observed, signifying an overrepresentation of players born at the end of the year. In the editions of the 1950s, a medium effect is identified, accompanied by a significant underrepresentation of athletes born in the second trimester of the year and an overrepresentation of those born in the fourth trimester. This overrepresentation in the fourth trimester was consistently observed and maintained throughout the 1960s.

**Figure 1:**
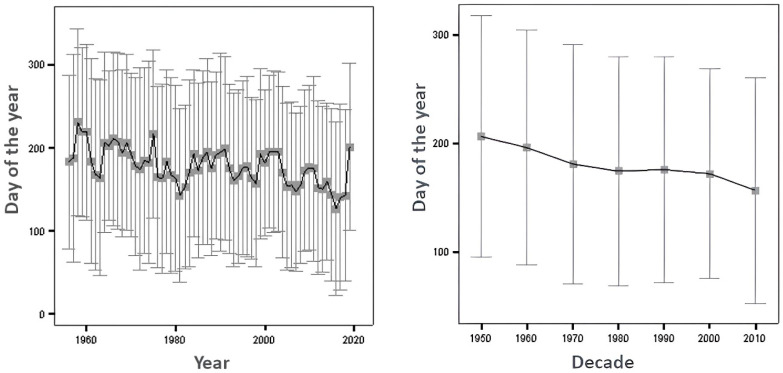
Evolution of the day of the year of birth: by year and by decade. Intervals show the mean plus/minus 1 standard deviation. Dots/lines show averages.

The previously observed trend of an inverse RAE in the 1950s and 1960s disappeared in the subsequent decades, with no significant RAE found. However, in the 2000s and 2010s, a significant RAE reappeared, characterized by a clear overrepresentation of players born in the first months of the year. A medium effect is identified in the editions from 2010 to 2020, marked by a significant overrepresentation of athletes born in the first quarter of the year and an underrepresentation of those born in the last half of the year (Table 1 and Figure 1).

The odds ratios (ORs) results reveal large effects for players born in S1 and S2 in the 2010s decade, indicating an overrepresentation of players born in the first half of the year. Medium OR effects are observed for players born in Q1 and Q2 in the 1950s, signifying an overrepresentation of players born in the first trimester of the year and an underrepresentation of those born in the second trimester. In the 2010s, medium OR effects are also found for Q1 and Q3, as well as Q1 and Q4, indicating an overrepresentation of players born in the first trimester and an underrepresentation of those born in the third and fourth trimesters, respectively. In the 2000s, a medium effect is observed for S1 and S2, signaling an overrepresentation of players born in the first half of the year and an underrepresentation of those born in the second half. Small effect sizes are identified in ORs for players born in Q1 and Q2 across overall, 1960s, 1970s, 1980s, and 2010s, indicating an overrepresentation of players born in the first trimester and an underrepresentation of those born in the second trimester. Similar small effects are found in Q1 and Q3 across overall, 1980s, and 1990s, and in Q1 and Q4 across overall, 1970s, 1980s, 1990s, 2000s, and 2020s, suggesting overrepresentation in the first trimester and underrepresentation in the third and fourth trimesters, respectively. Additionally, small effect sizes are observed in S1 and S2 across overall, 1980s, and 1990s (Table 2).

## 4. Discussion

Our main objective was to determine the existence of a relative age effect in soccer players nominated for the Ballon d’Or throughout its history. A significant RAE was identified in the overall sample, revealing three distinct phases. The initial phase (1950s, 1960s) manifested an inverse RAE. In the second phase (1970s, 1980s, 1990s), the RAE was not statistically significant. The third and more recent phase (2000s, 2010s) exhibited a highly pronounced and significant RAE.

A limited number of studies delve into the RAE among athletes receiving individual awards or nominations. Notably, Ford and Williams [35] conducted a study involving 205 professional athletes in soccer, ice hockey, baseball, and Australian soccer, revealing no evidence of RAE. These findings suggest a diminishing RAE among higher-level athletes, potentially explained by relatively younger athletes compensating for developmental disadvantages through enhanced technical skills. The awards investigated in this study were Most Valuable Player (MVP) or similar distinctions, spanning from 1987 to 2007. Although the sample size in this study (205 players) is considerably smaller than in the present study (1899 Ballon d’Or nominated players), results align in the 1980s and 1990s, indicating no RAE. However, a notable divergence occurs in the 2000s, where our study reveals a significant RAE. In contrast to the Ford and Williams study, the present research demonstrates that relatively younger players in our sample, particularly those born in the last quarters of the year, face discrimination. Importantly, the RAE not only persists in elite tournaments organized by FIFA, as highlighted by Saavedra-Garcia et al. [17], but also endures among players honored with the Ballon d’Or. This emphasizes the nuanced and evolving nature of RAE within different contexts and levels of competition, challenging the notion of its uniformity across all elite athletes.

While our study did not reveal a relative age effect among top-ranked players in the Ballon d’Or, it is noteworthy that in badminton, lower categories demonstrated a significant RAE between 2014 and 2018, particularly in players winning medals at European championships [36]. This finding, despite the different contexts and age groups analyzed in our study (senior category), aligns with the established trend that RAE tends to be more consistent in lower categories [37] both in male sports [3,6,11,14,38] and in female sport [6,8], although in the female category, this trend is less consistent.

Similarly, observations from the first Winter Youth Olympic Games in 2012, involving 557 athletes, indicated an overrepresentation of relatively older medal winners compared to those born at the end of the second eligible year [39]. This aligns with our study’s findings in the corresponding decade, even though we are comparing with athletes aged 15 to 19. The age categorization in our study prompts an intriguing question about whether the players, when participating in these categories, were in their first or second year of eligibility. The disparity in the likelihood of winning a medal across quarters mirrors the patterns identified in our research.

Further supporting evidence comes from the 12th Winter European Youth Olympic Festival in 2015 [40], where an overrepresentation of medalists born in the first quarter of the year was observed in lower categories. This two-year category study consistently showed a higher percentage of medalists born in the first quarter, aligning with the differences found in our study.

These cross-sport and cross-category comparisons underscore the multifaceted nature of RAE, influenced by factors such as age categories, sports, and competition levels. Understanding these nuances enhances the broader comprehension of the RAE’s impact on athletes’ success in various contexts.

The rare longitudinal studies on RAE make noteworthy contributions to the understanding of this phenomenon. In soccer corroborating our findings, an analysis spanning nine seasons in U7 to U18 categories uncovered an asymmetry in birth dates among both Premier League Academy and grassroots players. Particularly striking was the significantly higher RAE observed in higher-level players (Premier League Academy). Longitudinal analysis further revealed that the cumulative probability of remaining in the academy was higher for players born early in the year compared to those born later in the year of birth [38]. This longitudinal evidence provides robust support for the persistence of RAE in elite soccer. Our data are also in accordance with other longitudinal studies on soccer players participating in FIFA-organized competitions over a period exceeding one hundred years, the consistent presence of RAE across different levels of competition underscores its enduring influence on player development and selection processes, emphasizing the need for continued awareness and mitigation strategies in elite sports [17].

An examination of RAE in Spanish U12 basketball players throughout the decade from 2009 to 2018 revealed a higher participation of players born in the first half of the year. Notably, the top-ranked teams displayed a notable trend of having more players born in the first half of the year. This suggests that U12 basketball teams might experience improved performance due to the RAE [41]. Contrastingly, other studies propose varying patterns of RAE in basketball. Some contend that RAE exists in lower categories and diminishes to the point of disappearance in elite sports at the FIBA level [42]. However, in soccer, the disappearance of RAE is not evident, persisting at the youth and senior national team levels [3,7] as well as among athletes nominated for the Ballon d’Or.

An intriguing longitudinal study on Spanish handball players, conducted between 2005 and 2020, explored the RAE in U19 and U21 national teams, as well as senior national teams. The findings revealed a distinct pattern in U19 and U21 players, where a continuous decrease in the number of players was observed across the eight quarters representing two years of eligibility. In contrast, the senior national team exhibited a less pronounced decline, with a significant rebound in the fourth quarter and notably low representation of players born in the second quarter. Despite this, statistical significance did not align with the consistent existence of RAE [43]. This handball study presents a departure from the patterns observed in FIFA national soccer teams [17] and our study on the Ballon d’Or. While Q4 displayed higher frequencies than Q2 and Q3, it did not reach the overrepresentation observed in Q1. The inverse RAE detected in the 1950s and 60s, explained by the “underdog hypothesis”, posits that athletes born at the end of the year benefit from training with relatively older peers during development, aiding their transition from junior to elite sports [44,45]. However, this hypothesis diminishes in later decades. Talent identification and development programs, heavily influenced by RAE, may contribute to the dropout of unselected athletes from the sport [46]. This, coupled with the underdog hypothesis, provides potential explanations for the results found in the history of the Ballon d’Or. Moreover, the overrepresentation of players born in the first months of the year could potentially be even more pronounced.

The limited number of studies spanning multiple seasons or examining RAE over an extended period makes this research noteworthy. In the context of the National Hockey League, a study utilizing comprehensive data on every player in NHL history identified a significant RAE. However, despite the substantial sample size, the study did not delve into the evolution of RAE over time. Similarly, another NHL study by Deaner et al. [47] analyzed more than 25 years of RAE in NHL drafts but did not explore its temporal evolution.

In the realm of soccer, a study on the Bundesliga by Cobley et al. [48] evaluated RAE from the 1963–64 to the 2006–07 season, revealing a progressive increase in the effect of relative age among players born between 1950 and 1990. This aligns closely with our findings, suggesting a pronounced and escalating RAE over time. Furthermore, an analysis of RAE in soccer players participating in FIFA competitions from 1908 to 2012 established the existence of a dynamic and complex RAE concerning player age and the year of the competition in male FIFA competitions. This further emphasizes the evolving nature of RAE across different historical periods in soccer [17].

The main limitation of this study is that 63 countries are included, some of which do not even exist today (USSR, Yugoslavia, GDR, FRG, Czechoslovakia), as well as players with double and even triple nationality (up to 21 times). This makes it impossible to address the four quarters of the year in an orderly manner based on the cutoff date. In sports, the cutoff is set as the 1st of January in most of the competitions, so players born in the first months of the year have improved prospects in sports. For this reason, and being aware that a minimum bias may be generated, the January 1 cutoff date is the only way to approach a study of this type and is commonly used in the literature.

The present research contributes significantly to the understanding of RAE in sports and highlights the need for longitudinal analyses to capture the nuanced changes in RAE patterns over time.

## 5. Conclusions

In analyzing the history of the Ballon d’Or, several key patterns regarding relative age effect emerge:

Existence of relative age effect: RAE is evident and statistically significant among soccer players nominated for the Ballon d’Or throughout its history.

Top positions immunity: Interestingly, no RAE is found among players occupying the top positions in the Ballon d’Or awards. This suggests a unique dynamic or set of factors influencing the RAE pattern among the highest-performing players.

Evolution over time: The evolution of RAE over time exhibits variability. In the initial decades following the establishment of the Ballon d’Or, an inverse and significant RAE is observed. This is followed by a period where RAE seems to disappear, with no observable effect. However, in the most recent decades, RAE has resurfaced with increasing prominence.

These nuanced findings underscore the complexity of RAE in the context of soccer and prestigious individual awards like the Ballon d’Or. The historical perspective provides valuable insights into the dynamic nature of RAE, suggesting that its impact may be influenced by factors that evolve over time. The absence of RAE among top-ranking players adds an intriguing dimension, inviting further exploration into the unique circumstances that may contribute to this phenomenon at the highest levels of the sport.

## Figures and Tables

**Table 1:** Relative age effect in a global way and by decade.

	n	χ^2^	*p*-Value	V	Frequencies				Standardized Residuals			
					Q1	Q2	Q3	Q4	Q1	Q2	Q3	Q4
Overall	1899	14.88	0.002 *	0.051 ^s^	537	465	432	465	3.14 **	−0.36	−2.12 **	−0.62
Top 10	695	2.59	0.459	0.037 ^s^	187	175	172	161	1.17	0.15	−0.24	−1.07
Top 3	203	3.01	0.390	0.070 ^s^	51	57	54	41	0.12	0.91	0.40	−1.42
Top 1	66	0.75	0.861	0.061 ^s^	15	19	18	15	−0.38	0.57	0.27	−0.46
1950s	99	18.58	0.001 *	0.250 ^m^	23	12	22	42	−0.29	−2.54 **	−0.59	3.41 **
1960s	294	16.19	0.001 *	0.136 ^s^	70	58	63	103	−0.31	−1.78	−1.29	3.36 **
1970s	284	3.88	0.275	0.067 ^s^	81	59	75	69	1.36	−1.58	0.47	−0.25
1980s	300	7.45	0.059	0.091 ^s^	94	67	73	66	2.31 **	−0.89	−0.30	−1.10
1990s	297	3.21	0.360	0.060 ^s^	84	78	68	67	1.24	0.47	−0.79	−0.91
2000s	277	11.25	0.010	0.116 ^s^	74	88	64	51	0.67	2.29 **	−0.69	−2.25 **
2010s	258	25.31	0.000 *	0.183 ^m^	89	78	41	50	3.17 **	1.72	−2.98 **	−1.86
2020s	90	2.21	0.530	0.090 ^s^	22	25	26	17	−0.05	0.55	0.70	−1.19

* χ^2^ test statistical significance. V = Cramer’s V effect size (s: small; m: medium). Q1 to Q4 = trimester 1 to 4. ** Standardized residuals signification.

**Table 2:** Odds ratios with 95% confidence intervals: birthdate distributions between trimesters.

	Q1 vs. Q2	Q1 vs. Q3	Q1 vs. Q4	S1 vs. S2
Overall	1.22 (1.05–1.41) ^s^	1.34 (1.16–1.55) ^s^	1.22 (1.05–1.41) ^s^	1.25 (1.10–1.42) ^s^
1950s	2.19 (1.02–4.70) ^m^	1.06 (0.54–2.06)	0.41 (0.22–0.76)	0.30 (0.17–0.54)
1960s	1.27 (0.86–1.88) ^s^	1.15 (0.78–1.69)	0.58 (0.40–0.83)	0.59 (0.43–0.82)
1970s	1.52 (1.04–2.24) ^s^	1.11 (0.77–1.61)	1.25 (0.86–1.82) ^s^	0.95 (0.68–1.31)
1980s	1.59 (1.10–2.29) ^s^	1.42 (0.99–2.03) ^s^	1.62 (1.12–2.33) ^s^	1.34 (0.97–1.85) ^s^
1990s	1.11 (0.77–1.59)	1.33 (0.92–1.92) ^s^	1.35 (0.93–1.96) ^s^	1.44 (1.04–1.99) ^s^
2000s	0.78 (0.54–1.13)	1.21 (0.82–1.78)	1.62 (1.08–2.42) ^s^	1.98 (1.42–2.78) ^m^
2010s	1.22 (0.84–1.76) ^s^	2.79 (1.83–4.25) ^m^	2.19 (1.47–3.27) ^m^	3.37 (2.35–4.83) ^l^
2020s	0.84 (0.43–1.64)	0.80 (0.41–1.54)	1.39 (0.68–2.84) ^s^	1.19 (0.67–2.14)

Odds ratio thresholds (s: small; m: medium; l: large).

## Data Availability

Data are public on the internet Ballon d’Or website.
